# Three lignicolous freshwater fungi of *Neohelicomyces* (Tubeufiales, Tubeufiaceae) from Guizhou Province, China

**DOI:** 10.3897/mycokeys.134.188780

**Published:** 2026-06-17

**Authors:** Li-Juan Zhang, Hong-Xiu Liu, Xing-Juan Xiao, Xiang-Fu Liu, Ning-Guo Liu, Yong-Zhong Lu

**Affiliations:** 1 School of Food and Pharmaceutical Engineering, Guizhou Institute of Technology, Guiyang 550025, China Guizhou Key Laboratory of Agricultural Microbiology, Guizhou Academy of Agricultural Sciences Guiyang China; 2 Guizhou Key Laboratory of Agricultural Microbiology, Guizhou Academy of Agricultural Sciences, Guiyang 550009, China Center of Excellence in Fungal Research, Mae Fah Luang University Chiang Rai Thailand; 3 Center of Excellence in Fungal Research, Mae Fah Luang University, Chiang Rai 57100, Thailand School of Science, Mae Fah Luang University Chiang Rai Thailand; 4 School of Science, Mae Fah Luang University, Chiang Rai 57100, Thailand College of Biological Science and Food Engineering, Southwest Forestry University Kunming China; 5 College of Biological Science and Food Engineering, Southwest Forestry University, Kunming 650224, China Modern Industry School of Edible Fungi, Southwest Forestry University Kunming China; 6 Modern Industry School of Edible Fungi, Southwest Forestry University, Kunming 650224, China School of Food and Pharmaceutical Engineering, Guizhou Institute of Technology Guiyang China

**Keywords:** 1 new record, 2 new species, phylogeny, taxonomy, Tubeufiaceae

## Abstract

Lignicolous freshwater fungi are key decomposers in freshwater ecosystems, playing crucial roles in nutrient cycling and energy flow. During investigations into microfungi in freshwater habitats in Guizhou Province, China, six specimens of *Neohelicomyces* were collected from submerged decaying wood. Detailed morphological examinations, together with multi-gene phylogenetic analyses based on LSU, ITS, *rpb*2 and *LSU*1-α sequence data, were conducted to clarify their taxonomic placements. Phylogenetic results revealed that these collections represent two novel species, *N. brevis* and *N. wujiangensis*, and one known species, *N. astrictus*, which is reported here as a new habitat record from a freshwater habitat. The two new species can be distinguished from their closest relatives by differences in conidiophore morphology, conidial size and septation, and colony characteristics, and are well supported as independent lineages in the phylogenetic analyses. This study expands the species diversity of *Neohelicomyces* and highlights freshwater ecosystems in southwestern China as important reservoirs of helicosporous hyphomycetes diversity.

## Introduction

Lignicolous freshwater fungi represent a highly diverse assemblage of fungi that colonizes submerged woody debris in freshwater habitats, including streams, rivers, ponds, lakes, tree hollows, peat swamps, and dams (Hyde 1994; Wong et al. 1998; Luo et al. 2004, 2017, 2019; Pinruan et al. 2007; Hu et al. 2010; Hyde et al. 2016a, 2016b; Dong et al. 2020; Shen et al. 2022; Wang et al. 2024; Xu et al. 2025). These fungi are predominant members of Ascomycota and are mainly classified within Dothideomycetes and Sordariomycetes, with fewer representatives in Eurotiomycetes and Orbiliomycetes, as well as rare basidiomycetous taxa such as *Aegeritina tortuosa*, *Mycocalia reticulata*, and *Psathyrella aquatica* (Hyde and Goh 1998, 1999; Shearer et al. 2009; Swe et al. 2009; Jones et al. 2014; Lu et al. 2018a; Sun et al. 2019; Calabon et al. 2023). Ecologically, lignicolous freshwater fungi encompass endophytic, parasitic, pathogenic, saprobic, and mutualistic taxa (Shearer et al. 2007; Hyde et al. 2016a, 2016b; Sun et al. 2019; Shen et al. 2022; Calabon et al. 2023; Xu et al. 2025). In addition, they play an important role in freshwater ecosystems by decomposing woody substrates, thereby contributing to nutrient and carbon cycling, facilitating the release of organic matter, and supporting diverse aquatic life forms (Wong et al. 1998; Bucher et al. 2004; Jones et al. 2014; Wurzbacher et al. 2014; Hyde et al. 2016a, 2016b; Calabon et al. 2022, 2023).

Luo et al. (2017) established the *Neohelicomyces* based on molecular and morphological evidence, with *N. aquaticus* designated as the type species, which was collected from submerged decaying wood substrates in Yunnan Province, China. Morphologically, *Neohelicomyces* is characterized as a helicosporous hyphomycete; conidiophores are mononematous, macronematous, erect, branched or unbranched, and long or short; conidiogenous cells are monoblastic to polyblastic, integrated, and terminal or intercalary; conidia are acropleurogenous or pleurogenous, helicoid, hyaline, and guttulate (Luo et al. 2017; Lu et al. 2018b; Tibpromma et al. 2018; Crous et al. 2019a, 2019b; Lu et al. 2022). The sexual morph is characterized by ascomata superficial, solitary to scattered, subglobose, reddish-brown to brown; 8-spored, bitunicate asci, narrowly cylindrical, hyaline to pale brown, septate, roughened ascospores (Sun et al. 2025). According to Index Fungorum (https://www.indexfungorum.org/Names/Names.asp; on 13 January 2026), *Neohelicomyces* contains 42 species. In addition to its taxonomic diversity, recent investigations into secondary metabolites of *Neohelicomyces* have revealed that two compounds isolated from *N. hyalosporus* (PF11-1) exhibit moderate cytotoxic activity against human cancer cell lines, suggesting that members of this genus may represent a promising source of bioactive compounds with potential anti-tumor applications (Zhang et al. 2023).

In this study, six *Neohelicomyces* specimens were collected from freshwater habitats in Guizhou Province, China. Based on morphological observations, illustrations, and multi-gene phylogenetic analyses of LSU, ITS, *rpb*2, and *LSU*1-α, we describe two novel species, *N. brevis* and *N. wujiangensis*, and report *N. astrictus* as a new habitat record from freshwater environments.

## Materials and methods

### Sampling and examination of specimens

Specimens were collected from submerged decaying wood in freshwater habitats in Guizhou Province, China. Six samples of *Neohelicomyces* were collected in Guizhou province viz. GZAAS 25-07820 and GZAAS 25-07821 from the Zhenyuan County, Qiandongnan Miao and Dong Autonomous Prefectur, 27°14'40"N, 108°18'30"E, at 625 m above sea level, 3 May 2025; GZAAS 25-07822 (holotype) and GZAAS 25-07823 (isotype) from the Libo County, Qiannan Buyei and Miao Autonomous Prefecture, 25°17'6"N, 108°4'27"E, at 470 m above sea level, 31 May 2025; GZAAS 25-07824 (holotype) and GZAAS 25-07825 (isotype) from the Bijie City, Guizhou Province, China, 26°48'19"N, 106°6'9"E, at 940 m above sea level, 21 July 2025. Samples were placed in moistened plastic bags and transported to the laboratory, where they were incubated under moist conditions for 1–2 weeks prior to detailed examination. Fresh materials were dissected and observed for morphological characteristics, including the structures of conidiophores, conidiogenous cells, and conidia, using stereomicroscopes (SMZ 745 and SMZ 800N, Nikon, Tokyo, Japan) and an ECLIPSE Ni compound microscope (Nikon, Tokyo, Japan). Measurements were obtained with Tarosoft (R) Image Framework software, and photo-plates were assembled using Adobe Photoshop 2025 (version 26.5; Adobe Systems, CA, USA).

Single-spore isolations were carried out following the methodology described by Senanayake et al. (2020). Pure cultures were maintained at 28 °C under continuous illumination, and colony characteristics were examined and documented in detail. Dried specimens were deposited in the herbarium of the Guizhou Academy of Agriculture Sciences (GZAAS), Guiyang, China, while living cultures were preserved in the Guizhou Culture Collection (GZCC), Guiyang, China. Index Fungorum and Facesoffungi registration numbers (Jayasiri et al. 2015) were assigned to each taxon. The new fungal taxa were established following the guidelines outlined by Chethana et al. (2021).

### DNA extraction, PCR amplification, and sequencing

Actively growing mycelium was collected from PDA cultures after two weeks of incubation using sterile toothpicks and transferred into 1.5 mL microcentrifuge tubes. Total genomic DNA was isolated using the Ezup Column Fungi Genomic DNA Purification Kit (Sangon Biotech, Shanghai, China) in accordance with the manufacturer’s protocol. Four genetic markers, the internal transcribed spacer (ITS) with the primer pair of ITS4/ITS5 (White et al. 1990), large subunit rDNA (LSU) with the primer pair of LR0R/LR5 (Vilgalys and Hester 1990), translation elongation factor 1-alpha (*LSU*1-α) with the primer pair of EF1-983F/EF1-2218R (Carbone and Kohn 1999), and the RNA polymerase II second largest subunit (*rpb*2) with the primer pair of fRPB25F/fRPB2-7cR (Liu et al. 1999).

PCR amplifications were carried out in 25 μL reaction mixtures containing 21 μL of 1.1 × T3 Super PCR Mix (Tsingke Biotechnology Co., Ltd., Chengdu, China), 1 μL of each primer, and 2 μL of genomic DNA. Amplifications were performed on a JS-G9612 thermal cycler (Shanghai Peiqing Technology Co., Ltd., Shanghai, China). Cycling conditions for LSU, ITS, *rpb*2, and *LSU*1-α followed the protocols described by Ma et al. (2023). PCR products were examined by electrophoresis on 1% agarose gels, and subsequently purified and sequenced by Tsingke Biotechnology Co., Ltd. (Chengdu, China). All newly generated sequences were deposited in GenBank (https://ncbi.nlm.nih.gov/WebSub/).

### Phylogenetic analyses

Raw sequence chromatograms were inspected using BioEdit v.7.0.5.3 (Hall 1999) for quality control, including the identification of base-calling errors, ambiguous sites, and potential contamination. Consensus sequences were generated by assembling forward and reverse reads in SeqMan v.7.0.0 (DNASTAR, Madison, WI, USA; Swindell and Plasterer 1997). Reference sequences used in the phylogenetic analyses were retrieved from GenBank (Table 1) via the One-click Fungal Phylogenetic Tool (OFPT) (Zeng et al. 2023). Individual gene regions were aligned using the online MAFFT v.7 server, after which the resulting alignments were trimmed and manually refined with trimAl (-gt -gapthreshold < 0.6>) (Capella-Gutiérrez et al. 2009; Katoh and Standley 2013). The concatenated multi-gene dataset was assembled using SequenceMatrix v.1.7.8 (Vaidya et al. 2011). Phylogenetic analyses were conducted using maximum likelihood (ML) in RAxML-HPC on XSEDE via the CIPRES web portal under the GTRGAMMA substitution model with 1,000 bootstrap replicates. The best models were TIM3e+I+G4 for LSU, TVM+F+I+G4 for ITS, TNe+I+G4 for *rpb*2, and TIM+F+R3 for *LSU*1-α. unalysis was conducted using MrBayes v.3.2.7a on the CIPRES (Ronquist et al. 2012). Posterior probabilities (BYPP) were estimated using a Markov Chain Monte Carlo (MCMC) approach. Four Markov chains were run for 5,000,000 generations, and convergence was assessed using a stop rule with a standard deviation of split frequencies of 0.01 (http://www.phylo.org/portal2/, Accessed 29 December 2025).

**Table 1.** The table below lists the taxa used in this study, with their respective GenBank accession numbers.

**Taxon**	**Strain**	**GenBank Accessions**			
		**ITS**	**LSU**	***LSU*1-α**	***rpb*2**
*Muripulchra aquatica*	KUMCC 15-0276	KY320534	KY320551	KY320564	MH551058
*M. aquatica*	MFLUCC 15-0249^T^	KY320532	KY320549	—	—
*Neohelicomyces acropleurogenus*	CGMCC 3.25549^T^	PP626594	PP639450	PP596351	PP596478
*N. aquaticus*	KUMCC 15-0463	KY320529	KY320546	KY320562	MH551065
*N. aquaticus*	MFLUCC 16-0993^T^	KY320528	KY320545	KY320561	MH551066
*N. aquisubtropicus*	GZCC 23-0080^T^	PQ098499	PQ098537	PV768327	PV768336
*N. aquisubtropicus*	GZCC 24-0163	PV730410	PV730414	PV768328	PV768337
*N. aseptatus*	CGMCC 3.25564^T^	PP626595	PP639451	PP596352	PP596479
***N. astrictus***	**GZCC 25-27619**	**PX723937**	**PX723943**	**PX733141**	**PX733130**
***N. astrictus***	**GZCC 25-27620**	**PX723938**	**PX723944**	**PX733142**	**PX733131**
*N. baochengensis*	GZCC 25-0669^T^	PX848697	PX848711	PZ095451	PZ095447
*N. baochengensis*	GZCC 25-0670	PX848698	PX848712	PZ095452	PZ095448
*N. astrictus*	HKAS 105122^T^	PQ898760	PQ898796	PV040811	—
***N. brevis***	**GZCC 25-27621^T^**	**PX723940**	**PX723948**	**PX733145**	**PX733134**
***N. brevis***	**GZCC 25-27622**	**PX723939**	**PX723947**	**PX733146**	**PX733135**
*N. brunneus*	HKAS 105147^T^	PQ898768	PQ898805	PV040818	—
*N. coffeae*	GMBCC 2225^T^	PX308843	PX308848	PX314510	PX314514
*N. coffeae*	GMBCC 2226	PX308844	PX308849	PX314511	PX314515
*N. davidii*	GZCC 24-0290^T^	PV820368	PV856184	—	—
*N. dehongensis*	MFLUCC 18-1029^T^	NR_171880	MN913709	MT954393	—
*N. denticulatus*	GZCC 19-0444^T^	OP377832	MW133855	—	—
*N. denticulatus*	GZCC 23-0073^T^	PP626596	PP639452	PP596353	PP596480
*N. denticulatus*	UAMH 10535	AY916462	AY856913	—	—
*N. deschampsiae*	CPC 33686^T^	MK442602	MK442538	—	—
*N. edgeworthiae*	CGMCC 3.25565^T^	PP626597	PP639453	PP596354	PP596481
*N. grandisporus*	KUMCC 15-0470^T^	KX454173	KX454174	—	MH551067
*N. guizhouensis*	GZCC 23-0725^T^	PP512969	PP512973	PP526727	PP526733
*N. guizhouensis*	GZCC 23-0726	PP512970	PP512974	PP526728	PP526734
*N. guttulatus*	CGMCC 3.25550^T^	PP626598	PP639454	PP596355	—
*N. guttulatus*	GZCC 23-0406	PP626599	PP639455	PP596356	PP596482
*N. hainanensis*	GZCC 22-2009^T^	OP508734	OP508774	OP698085	OP698074
*N. hainanensis*	GZCC 22-2027	OP508735	OP508775	OP698086	OP698075
*N. helicosporus*	GZCC 23-0633^T^	PP512971	PP512975	PP526729	PP526735
*N. helicosporus*	GZCC 23-0634	PP512972	PP512976	PP526730	PP526736
*N. hyalosporus*	GZCC 16-0086^T^	MH558745	MH558870	MH550936	MH551064
*N. hydei*	GZCC 23-0727^T^	—	PP512977	PP526731	PP526737
*N. hydei*	GZCC 23-0728	—	PP512978	PP526732	PP526738
*N. kevinianus*	GZCC 24-0073^T^	PV820369	PV856185	—	—
*N. lignicola*	CGMCC 3.25551^T^	PP626600	PP639456	PP596357	PP596483
*N. longisetosus*	NCYU 106H1-1-1^T^	MT939303	—	—	—
*N. macrosporus*	CGMCC 3.25552^T^	PP626601	PP639457	PP596358	PP596484
*N. maolanensis*	GZCC 23-0079^T^	—	PQ098529	PQ490683	PQ490677
*N. maolanensis*	GZCC 24-0148	—	PQ522500	PQ490682	PQ490676
*N. melaleucae*	CPC 38042^T^	MN562154	MN567661	MN556835	—
*N. melaleucae*	KUNCC 23-14314	PP664108	PP664112	—	—
*N. pallidus*	CBS 245.49	MH856510	—	—	—
*N. pallidus*	CBS 271.52	AY916461	AY856887	—	—
*N. pallidus*	CBS 962.69	AY916460	AY856886	—	—
*N. pandanicola*	KUMCC 16-0143^T^	MH275073	MH260307	MH412779	—
*N. polyblastus*	GZCC 24-0006^T^	PX278046	PX278040	PX717280	PX717287
*N. polyblastus*	GZCC 24-0007	PX278047	PX278041	PX717281	PX717288
*N. puerensis*	GMBCC 2217^T^	PQ737369	PX308846	PX314508	PX314512
*N. puerensis*	GMBCC 2218	PQ737370	PX308847	PX314509	PX314513
*N. qixingyaensis*	CGMCC 3.25569^T^	PP626602	PP639458	PP596359	PP596485
*N. saprobicus*	GZAAS 23-0826	PX625156	PX625152	—	—
*N. saprobicus*	GZAAS 23-0827	PX625157	PX625153	—	—
*N. sexualis*	HGUP 24-0021^T^	PQ570844	PQ570861	—	—
*N. subtropicus*	GZCC 23-0076^T^	PQ098492	PQ098530	PQ490685	PQ490679
*N. subtropicus*	GZCC 24-0147	PQ522498	PQ522501	PQ490684	PQ490678
*N. terrestricola*	GZCC 23-0402^T^	PX278048	PX278042	PX717282	PX717289
*N. terrestris*	GZCC 23-0399^T^	PX575639	PX575662	PX512845	PX512836
*N. thailandicus*	GZCC 23-0400	PP626603	PP639459	PP596360	PP596486
*N. thailandicus*	MFLUCC 11-0005^T^	NR_171882	MN913696	—	—
*N. tropicus*	GZCC 25-0661^T^	PX575641	PX575664	PX512847	PX512838
*N. ubmersus*	MFLUCC 16-1106^T^	KY320530	KY320547	—	MH551068
*N. uniramulosus*	GZCC 25-0750^T^	PX625158	PX625154	—	—
*N. uniramulosus*	GZCC 25-0751	—	PX625155	—	—
***N. wujiangensis***	**GZCC 25-27623^T^**	—	**PX723945**	**PX733143**	**PX733132**
***N. wujiangensis***	**GZCC 25-27624**	—	**PX723946**	**PX733144**	**PX733133**
*N. wuzhishanensis*	GZCC 23-0410^T^	PQ098494	PQ098532	PV768325	PV768334
*N. wuzhishanensis*	GZCC 24-0164	PV730409	PV730413	PV768326	PV768335
*N. xiangshuiensis*	GZCC 25-0671^T^	—	PX848699	PZ095453	PZ095449
*N. xiangshuiensis*	GZCC 25-0672	—	PX848700	PZ095454	PZ095450
*N. xiayadongensis*	CGMCC 3.25568^T^	PP626604	PP639460	PP596361	PP596487
*N. xiayadongensis*	MUCL 15702	AY916459	AY856873	—	—
*N. yunnanensis*	GZCC 23-0735^T^	PP664109	PP664113	—	—

Note: “T” represents the ex-type strain. “—” indicates data unavailability. Newly generated sequences are represented in bold.

The phylogenetic tree was visualized and edited using FigTree v1.4.0 (http://tree.bio.ed.ac.uk/software/figtree/), and the final figure design and layout were completed with Adobe Photoshop 2024 and Adobe Illustrator 2021 (Adobe Systems, San Jose, CA, USA).

## Results

### Phylogenetic analysis

**Figure 1.**
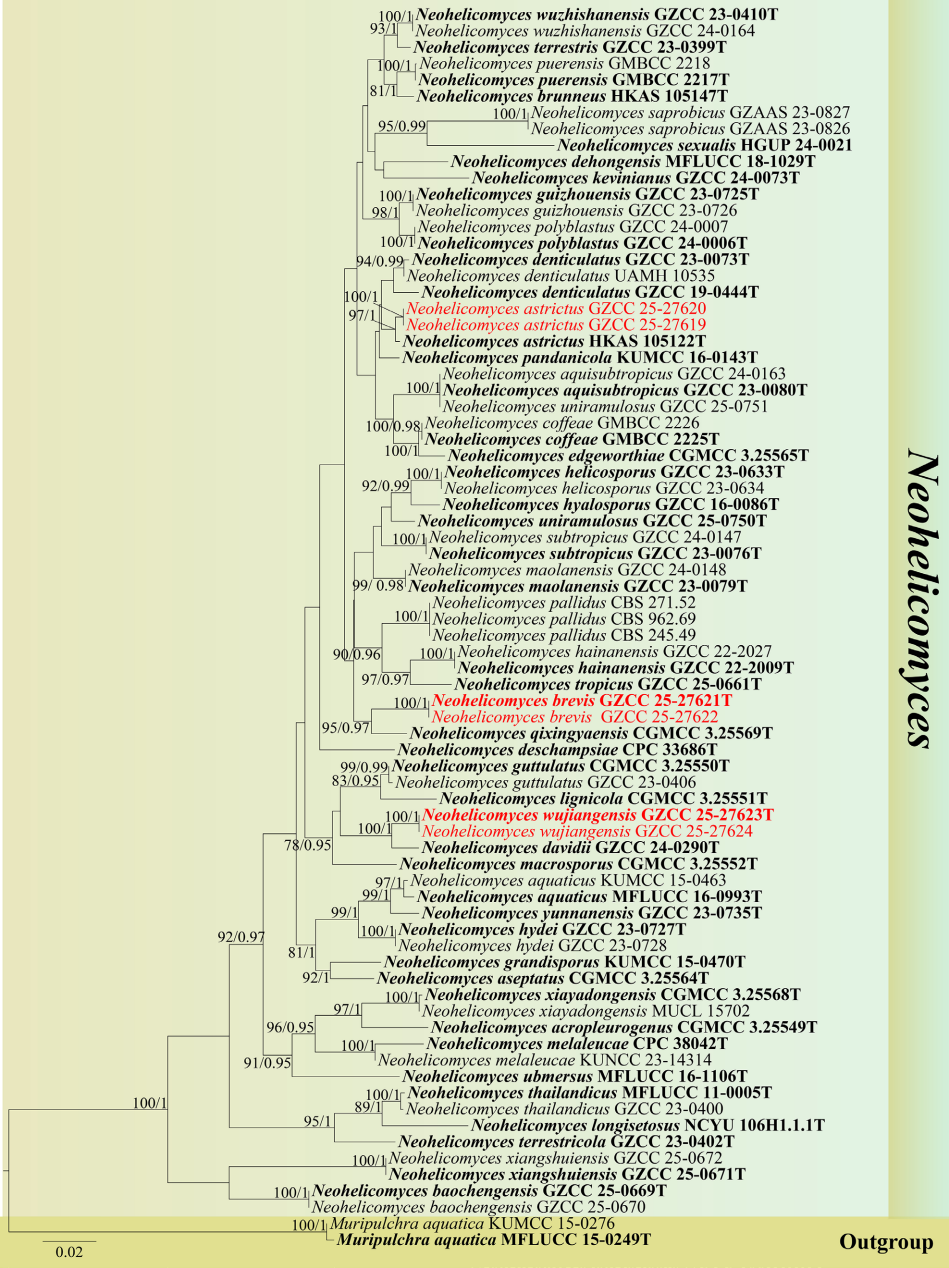
Maximum Likelihood majority rule consensus tree for the LSU, ITS, *rpb*2, and *LSU*1-α sequence data. ML bootstrap support values (ML ≥ 75%) and Bayesian posterior probabilities (BYPP ≥ 0.95) are indicated below or above the nodes. Ex-type strains are bold and marked with T, and the newly generated sequences are in red.

Partial nucleotide sequences of LSU, ITS, *rpb*2, and *LSU*1-α were used to infer the phylogenetic positions of the newly collected taxa. The combined dataset comprised sequences from 76 isolates, with *Muripulchra aquatica* (KUMCC 15-0276 and MFLUCC 15-0249) selected as the outgroup taxa. The concatenated sequence matrix includes ITS (1–533 bp), LSU (534–1,380 bp), *rpb*2 (1,321–2,425), and *LSU*1-α (2,426–3,326 bp). The best-scoring ML tree generated by RAxML (Fig. 1) had a final optimization likelihood value of -16070.228589. The estimated base frequencies were A = 0.249354, C = 0.246531, G = 0.257185, T = 0.246931; substitution rates AC = 1.151853, AG = 5.064548, AT = 2.728704, CG = 1.140121, CT = 9.056316, GT = 1.000000. The gamma distribution shape parameter alpha was estimated as 0.146752. The concatenated LSU, ITS, *rpb*2 and *LSU*1-α datasets were analyzed using ML and BI methods with similar tree topologies.

The topology of the ML and BI analysis results is consistent with the phylogenetic results of Zhang et al. (2026). The phylogenetic analyses showed that six newly obtained isolates were distributed among three taxa. GZCC 25-27620 and GZCC 25-27619 of *Neohelicomyces astrictus* clustered with the ex-type strain HKAS 10-5122, supported by 97% ML/1.00 BYPP (Fig. 1). Similarly, the strains of GZCC 25-27622 and GZCC 25-27621 of *N. brevis* formed a distinct clade sister to *N. qixingyaensis* (CGMCC 3.25569), supported by 95% ML/0.97 BYPP. In addition, the remaining GZCC 25-27624 and GZCC 25-27623 of *N. wujiangensis* formed a clade sister to *N. davidii* (GZCC 24-0290) (100% ML/1.00 BYPP).

### Taxonomy

#### Neohelicomyces astrictus

Taxon classificationFungiTubeufialesTubeufiaceae

C.G. Lin, K.D. Hyde & Jian K. Liu, Fungal Diversity [225] (2025)

95EFD75A-009E-56AF-8A89-DCD02DD7439B

Index Fungorum: IF903869

Facesoffungi Number: FoF17732

Fig. 2

##### Description.

***Saprobic*** on decaying wood in a freshwater habitat. **Sexual morph**: Undetermined. **Asexual morph**: Hyphomycetous, helicosporous. ***Colonies*** on natural substrate superficial, white, gregarious, glistening. ***Mycelium*** partly superficial, partly immersed, branched, septate, smooth, hyaline to pale brown, with masses of crowded, glistening conidia. ***Conidiophores*** 50–350 × 4–9 µm (x̄ = 200 × 6 μm, n = 20), macronematous, mononematous, cylindrical, unbranched, septate, thick-walled, hyaline to pale brown. ***Conidiogenous cells*** 10–17 × 3.5–9 µm (x̄ = 15 × 5 μm, n = 30), holoblastic, polyblastic, integrated, terminal and intercalary, cylindrical, smooth-walled, hyaline to pale brown, with denticles; denticles 1.4–4.4 × 0.9–2.6 µm (x̄ = 3.4 × 1.4 μm, n = 30), hyaline. ***Conidia*** 90–170 × 2–3.3 µm (x̄ = 121 × 2.6 μm, n = 30), helicoid, solitary, acropleurogenous, rounded at tip, 15–18 μm diam, tightly coiled 2–3 times, becoming loosely coiled in water, indistinctly multi-septate, guttulate, smooth-walled, hyaline.

**Figure 2.**
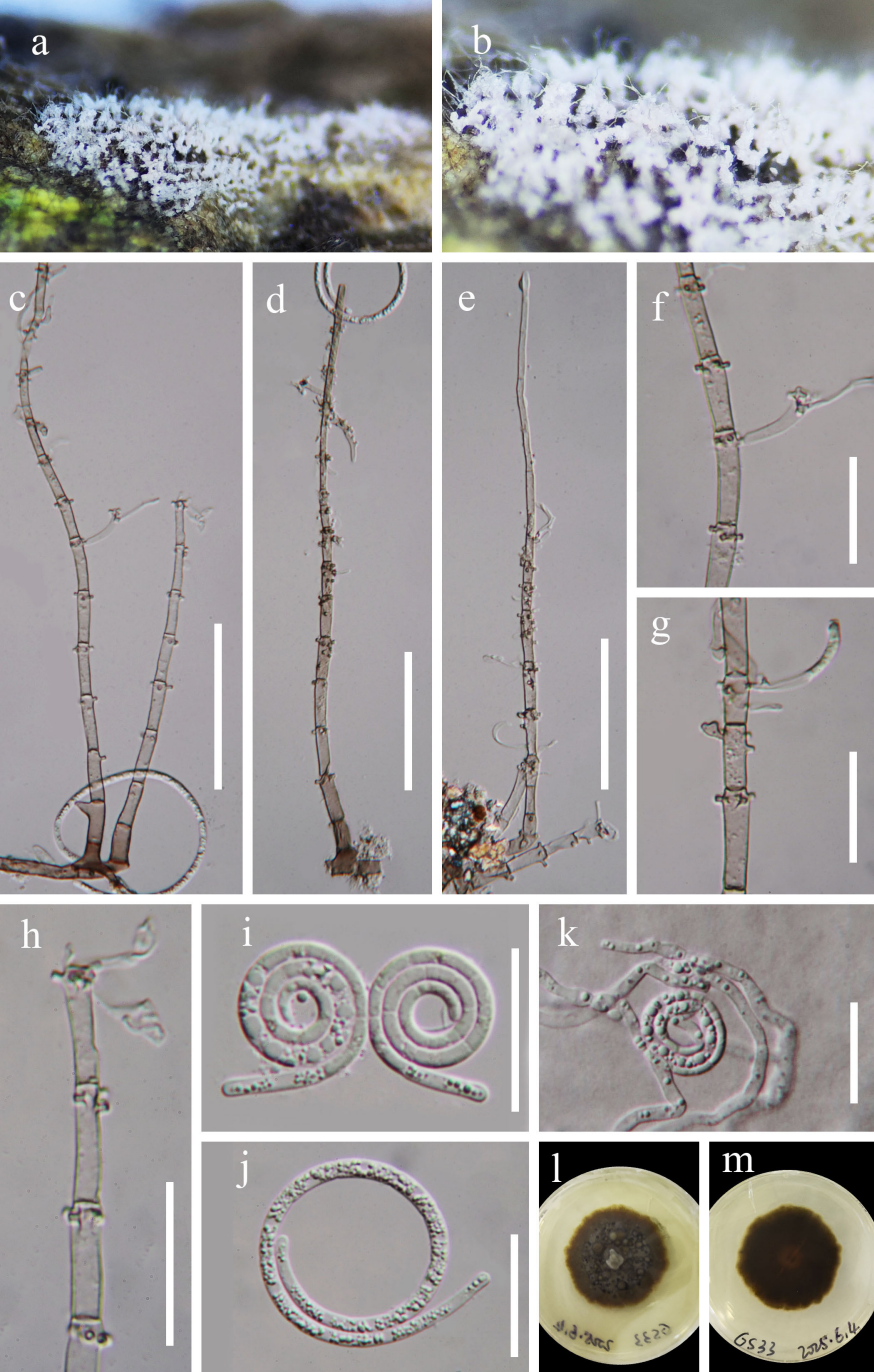
*Neohelicomyces astrictus* (GZAAS 25-07820, new habitat record). **a, b**. Colonies on the host surface; **c–e**. Conidiophores and conidia; **f–h**. Conidiogenous cells; **i, j**. Conidia; **k**. Germinated conidium; **l, m**. Colony on PDA (above and below). Scale bars: 50 μm (**c–e**); 20 μm (**f–k**).

##### Cultural characteristics.

Conidia germinated on PDA medium within 12 h and germ tube produced from conidia. *Colonies* on PDA medium reaching to 40 mm diam. after 43 days at 25 °C under natural light, dry, dense, with irregular shape, raised surface, undulated margin, brown to dark brown. The reverse is pale brown to dark brown, becoming paler near the center.

##### Material examined.

**China** • Guizhou Province, Qiandongnan Miao and Dong Autonomous Prefectur, Zhenyuan County, 27°14'40"N, 108°18'30"E, altitude 625 m, on submerged decaying wood in a stream, 3 May 2025, Xingjuan Xiao, GS33 (GZAAS 25-07820), living culture GZCC 25-27619; *ibid*. GS33b (GZAAS 25-07821), living culture GZCC 25-27620.

##### Notes.

Phylogenetic results showed that the new isolates GZCC 25-27619 and GZCC 25-27620 clustered with *Neohelicomyces astrictus* (HKAS 105122, ex-type), with support values of 97% ML/1.00 BYPP (Fig. 1). Pairwise nucleotide comparisons revealed that the new isolate GZCC 25-27619 is similar to HKAS 105122 in 479/486 bp (99%) of the ITS region, 764/764 bp (100%) of the LSU region, and 883/883 bp (100%) of the *LSU*1-αα region. Morphologically, our isolate is consistent with the holotype HKAS 10-5122 of *Neohelicomyces astrictus* in key diagnostic features, including helicoid, hyaline conidia and similar conidiogenous cell (Lin et al. 2025). However, it differs in having longer conidiophores (50–350 μm vs. 22–161 μm) and somewhat longer conidia (90–170 μm vs. 100–136.5 μm). Despite these minor differences, the overall morphology and phylogenetic placement support its identification as *N. astrictus*. Notably, our specimen was collected from a freshwater habitat, whereas HKAS 10-5122 was reported from a terrestrial habitat (Lin et al. 2025), representing a new habitat record for this species.

#### Neohelicomyces brevis

Taxon classificationFungiTubeufialesTubeufiaceae

L.J. Zhang, Y.Z. Lu & X.J. Xiao sp. nov.

E53EBDCF-EECC-5394-A530-64CE8FE467F5

Index Fungorum: IF904901

Facesofungi Number: FoF192203

Fig. 3

##### Etymology.

Referring to the short conidiophores of holotype.

##### Holotype.

GZAAS 25-07822.

##### Description.

***Saprobic*** on decaying wood in a freshwater habitat. **Sexual morph**: Undetermined. **Asexual morph**: Hyphomycetous, helicosporous. ***Colonies*** on natural substrate superficial, white, gregarious, glistening. ***Mycelium*** partly superficial, partly immersed, branched, aseptate, smooth, pale brown to brown, glistening conidia. ***Conidiophores***, micronematous. ***Conidiogenous cells*** 6–12 × 2–5 µm (x̄ = 10 × 3.5 μm, n = 20), holoblastic, monoblastic or polyblastic, integrated, cylindrical, smooth-walled, hyaline, denticulate, truncate at apex after conidial secession. ***Conidia*** 113–186 × 2–4 µm (x̄ = 145 × 3 μm, n = 30), helicoid, solitary, rounded at tip, 18–26 μm diam, tightly coiled 2 1/2–3 1/3 times, becoming loosely coiled in water, indistinctly multiseptate, guttulate, smooth-walled, hyaline.

##### Cultural characteristics.

Conidia germinated on PDA within 12 h, and germ tube produced from conidia. *Colonies* on PDA medium reaching to 35 mm diam. after 70 days at 25 °C under natural light, dry, dense, with irregular shape, raised surface, and filamentous margin. The outermost mycelium was immersed in the medium, brown to pale brown. The reverse is pale brown to dark brown, becoming paler near the center. No diffusible pigment was observed.

**Figure 3.**
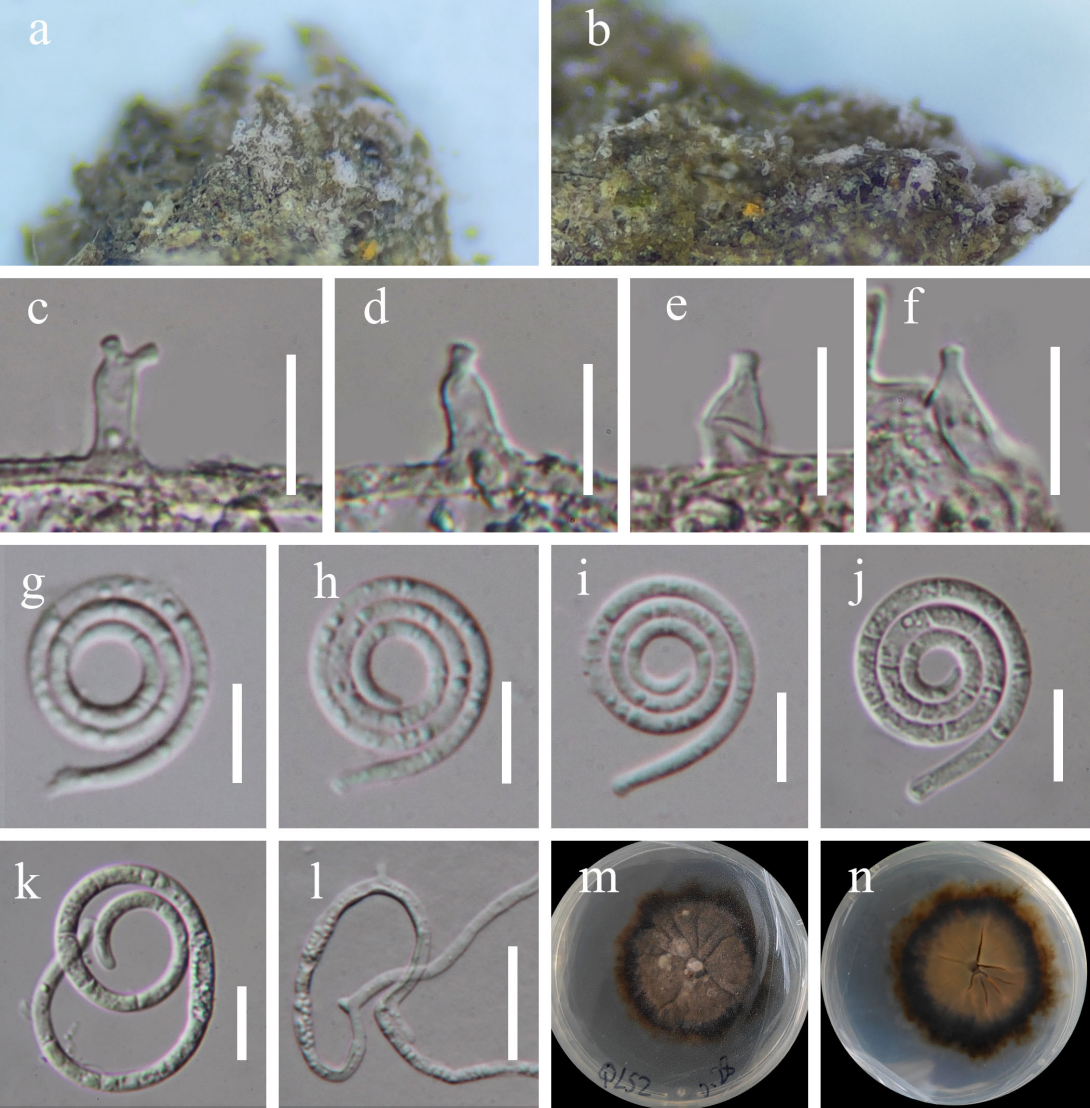
*Neohelicomyces brevis* (GZAAS 25-07822, holotype). **a, b**. Colonies on the host surface; **c–f**. Conidiophores and conidiogenous cells; **g–k**. Conidia; **l**. Germinated conidium; **m, n**. Colony on PDA (above and below). Scale bars: 20 μm (**l**); 10 μm (**c–k**).

##### Material examined.

**China** • Qiannan Buyei and Miao Autonomous Prefecture, Libo County, Guizhou Maolan National Nature Reserve, 25°17'6"N, 108°4'27"E, altitude 470 m, on submerged decaying wood in a stream, 31 May 2025, Xingjuan Xiao, QLS2 (GZAAS 25-07822, holotype), ex-type culture GZCC 25-27621; *ibid*. QLS2b (GZAAS 25-07823, isotype), ex-isotype culture, GZCC 25-27622.

##### Notes.

Phylogenetically, the two new strains GZCC 25-27621 and GZCC 25-27622 clustered into a distinct monophyletic clade, forming a sister clade to *Neohelicomyces qixingyaensis* (CGMCC 3.25569), with support values of 95% ML/0.97 BYPP (Fig. 1). Pairwise nucleotide comparisons revealed that *N. brevis* (GZCC 25-27621, ex-type) and *N. qixingyaensis* (CGMCC 3.25569, ex-type) shared 402/424 bp (95%) similarity in the ITS region, 787/788 bp (99%) in the LSU region, 915/948 bp (97%) in the *rpb*2 region, and 853/855 bp (98%) in the *LSU*1-α region, excluding gaps. Morphologically, *N. qixingyaensis* (HKAS 128928, holotype) possesses developed macronematous, mononematous conidiophores (94.5–187 × 3.5–6 μm), monoblastic or polyblastic, integrated conidiogenous cells with denticles (7–18 × 3–5.5 μm), and helicoid, aseptate conidia that do not become loose in water (98–147 μm × 1.5–3 μm) (Ma et al. 2024b). In contrast, our new isolate has micronematous conidiophores and produces conidia that become loosely coiled in water, are septate, and are longer than *N. qixingyaensis* (113–186 × 2–4 µm vs. 98–147 μm × 1.5–3 μm) (Ma et al. 2024b). The two species differ ecologically, with *N. brevis* being saprobic in freshwater habitats, whereas *N. qixingyaensis* occurs in terrestrial habitats (Ma et al. 2024b). Based on both molecular and morphological evidence, the new species *Neohelicomyces brevis* is herein proposed.

#### Neohelicomyces wujiangensis

Taxon classificationFungiTubeufialesTubeufiaceae

L.J. Zhang, Y.Z. Lu & X.J. Xiao sp. nov.

F850B521-8616-5B29-B448-58B19A1735C4

Index Fungorum: IF904902

Facesofungi Number: FoF192204

Fig. 4

##### Etymology.

Referring to the type locality (Wujiang River), where the holotype was collected.

##### Holotype.

GZAAS 25-07824.

##### Description.

***Saprobic*** on decaying wood in a freshwater habitat. **Sexual morph**: Undetermined. **Asexual morph**: Hyphomycetous, helicosporous. ***Colonies*** on natural substrate superficial, white, gregarious, glistening. ***Mycelium*** partly superficial, partly immersed, composed of branched, septate, smooth, pale brown to brown hyphae. ***Conidiophores*** 35–120 × 3–6.5 µm (x̄ = 70 × 4 μm, n = 20), macronematous, mononematous, erect, mostly simple, flexuous, cylindrical, hyaline to pale brown, septate, thick-walled. ***Conidiogenous cells*** holoblastic, monoblastic or polyblastic, integrated or discrete, sympodial, terminal or intercalary, cylindrical or repeatedly geniculate, smooth-walled, hyaline to brown, truncate at apex after conidial secession. ***Conidia*** 150–300 × 2–4 µm (x̄ = 230 × 3 μm, n = 30), helicoid, solitary, acropleurogenous, rounded at tip, 21–32 μm diam, tightly coiled 3^2^/_3_–4 times, becoming loosely coiled in water, indistinctly multiseptate, guttulate, smooth-walled, hyaline.

**Figure 4.**
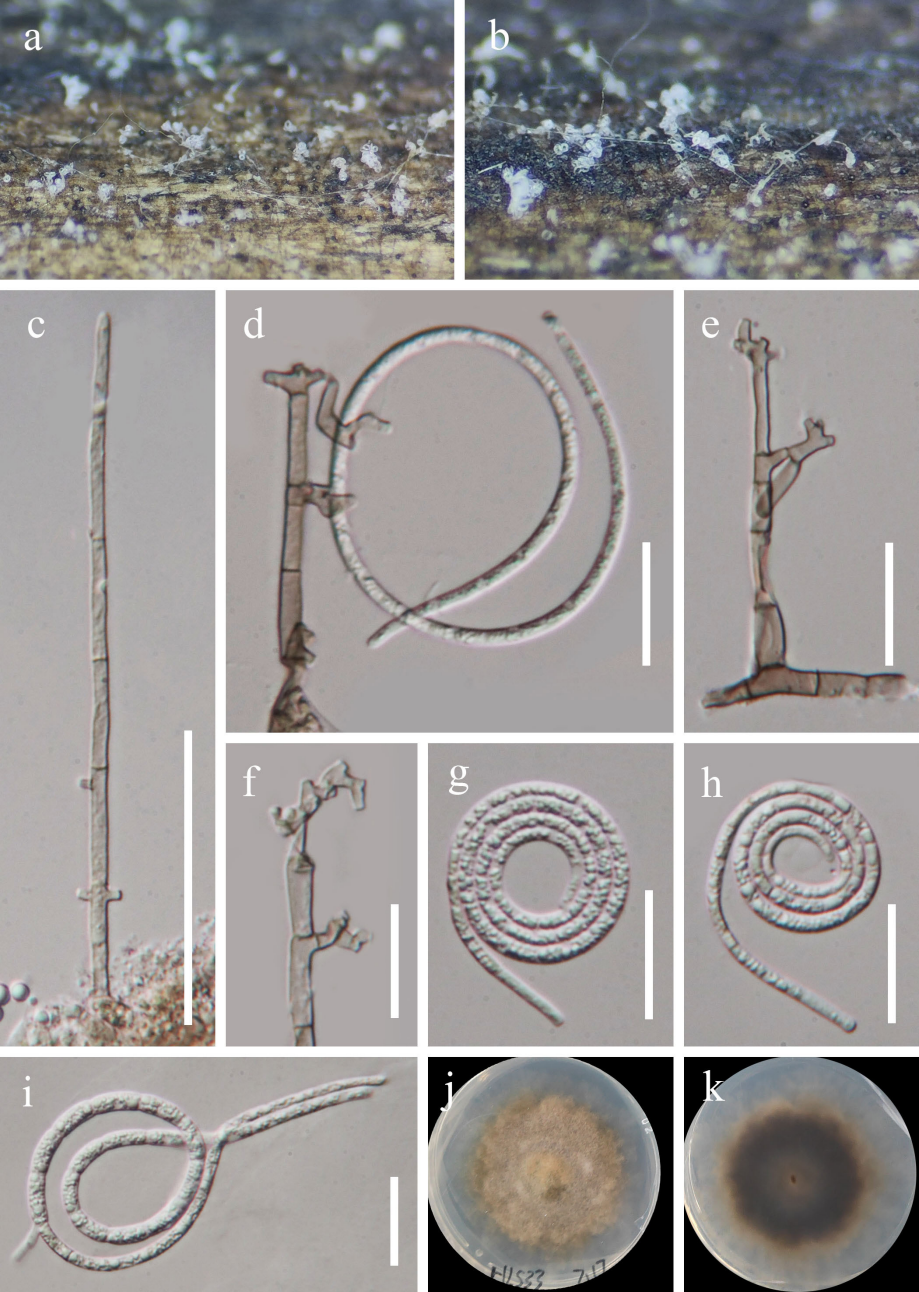
*Neohelicomyces wujiangensis* (GZAAS 25-07824, holotype). **a, b**. Colonies on the host surface; **c–f**. Conidiophores, conidiogenous cells and conidia; **g, h**. Conidia; **i**. Germinated conidium; **j, k**. Colony on PDA (above and below). Scale bars: 50 μm (**c**); 20 μm (**d–i**).

##### Cultural characteristics.

Conidia germinated on PDA within 12 h, and germ tube produced from conidial filaments. *Colonies* on PDA medium reaching to 25 mm diam. after 33 days at 28 °C under natural light, dry, dense, with irregular shape, raised surface, and filamentous margin, pale brown. The reverse is pale brown to dark brown, becoming paler near the center. No diffusible pigment was observed.

##### Material examined.

**China** • Guizhou Province, Bijie City, Wujiang river, 26°48'19"N, 106°6'9"E, altitude 940 m, on submerged decaying wood in Wujiang river, 21 July 2025, Xingjuan Xiao, HLS33 (GZAAS 25-07824, holotype), ex-type culture GZCC 25-27623; *ibid*. HLS33b (GZAAS 25-07825, isotype), ex-isotype culture, GZCC 25-27624.

##### Notes.

In the phylogenetic tree (Fig. 1), our two new strains GZCC 25-27623 and GZCC 25-27624 formed a distinct clade and were sister to *Neohelicomyces davidii* (GZCC 24-0290, ex-type), with support values of 100% ML/1.00 BYPP. Pairwise nucleotide comparisons showed that *N. wujiangensis* (GZCC 25-27623, ex-type) and *N. davidii* (GZCC 24-02907, ex-type) share 402/424 bp (95%) similarity in the ITS region, 778/779 bp (99%) in the LSU region, 1048/1066 bp (98%) in the *rpb*2 region, and 857/874 bp (98%) in the *LSU*1-α region, excluding gaps. Morphologically, the new isolate differs from *N. davidii* (HKAS 145875, holotype) by having shorter conidiophores (35–120 µm vs. 52–169 µm) and white colonies on the natural substrate, whereas those of *N. davidii* are white to pale pink (Xiao et al. 2025). In culture, *N. davidii* forms colonies with a rough surface and undulate margins, whereas *N. brevis* produces dense, dry colonies with a raised surface and filamentous margins (Xiao et al. 2025). Based on both molecular and morphological evidence, the new species *Neohelicomyces wujiangensis* is herein proposed.

## Discussion

The family Tubeufiaceae represents a distinctive lineage within Tubeufiales, predominantly characterized by helicosporous asexual morphs and a saprobic lifestyle, with diverse morphologies, habitats, and a worldwide distribution (Boonmee et al. 2014; Luo et al. 2017, 2019; Lu et al. 2018b; Dong et al. 2020; Ma et al. 2024a, 2024b, 2025). Integrative analyses combining morphology and multilocus datasets (ITS, LSU, *rpb*2, and *LSU*1*-α*) have substantially improved taxonomic resolution and revealed that the family is more diverse than previously recognized, although generic boundaries have historically been obscured by strong morphological convergence of helicosporous conidia and the rarity of sexual morphs (Linder 1929; Goos 1985, 1986, 1989; Tsui et al. 2006; Zhao et al. 2007; Boonmee et al. 2011, 2014, 2021; Hyde et al. 2016a, 2016b; Luo et al. 2017, 2019; Lu et al. 2018b, 2022; Tibpromma et al. 2018; Crous et al. 2019a, 2019b; Dong et al. 2020; Hsieh et al. 2021; Yang et al. 2023; Ma et al. 2024a, 2024b, 2025; Peng et al. 2025). To date, Tubeufiaceae encompasses more than 54 genera and 450 species (Hyde et al. 2024; Ma et al. 2024b). Ecologically, members of Tubeufiaceae are primarily saprobes on woody substrates, occurring in both freshwater and terrestrial habitats, with freshwater ecosystems representing important centers of diversification. Several taxa exhibit ecological plasticity by inhabiting both environments, suggesting adaptive flexibility within the family (Linder 1929; Goos 1989; Tsui et al. 2001; Zhao et al. 2007; Lu et al. 2018b; Yang et al. 2023; Ma et al. 2024b, 2025). Biogeographically, Tubeufiaceae species are widely distributed in tropical and subtropical regions, with China and Southeast Asia emerging as major diversity hotspots; however, this pattern is likely influenced by uneven sampling, indicating that global diversity remains underestimated (Luo et al. 2017, 2019; Dong et al. 2020; Lu et al. 2022; Yang et al. 2023; Ma et al. 2024a, 2024b, 2025; Xiao et al. 2025). Despite significant recent taxonomic progress, unresolved issues persist, including incomplete life-cycle connections, limited ecological and functional data, and insufficient molecular coverage for some historically described taxa. Within this framework, *Neohelicomyces*, characterized by rapid species accumulation, relatively comprehensive molecular sampling, and a broad ecological amplitude across freshwater and terrestrial habitats, provides a valuable model for elucidating diversification patterns and ecological adaptation within Tubeufiaceae.

*Neohelicomyces* currently comprises 42 species, including the two new species (*Neohelicomyces brevis* and *N. wujiangensis*), which are widely distributed across China, the Czech Republic, Germany, Italy, Japan, the Netherlands, Thailand, and the USA (Hsieh et al. 2021; Yang et al. 2023; Ma et al. 2024a, 2024b, 2025; Peng et al. 2025). Ecologically, members of *Neohelicomyces* are predominantly saprobic on decaying plant material, especially woody substrates, and are frequently collected from both freshwater and terrestrial habitats (Luo et al. 2017, 2019; Lu et al. 2018b; Tibpromma et al. 2018; Crous et al. 2019a, 2019b; Lu et al. 2022; Yang et al. 2023; Ma et al. 2024a, 2024b, 2025; Peng et al. 2025; Sun et al. 2025; Xiao et al. 2025). While some species show a preference for either submerged wood in freshwater environments or exposed plant debris in terrestrial settings, several taxa have been reported from both habitats, indicating a relatively broad ecological tolerance within the genus (Lu et al. 2018b; Ma et al. 2025). Notably, some species of *Neohelicomyces* from different habitats may exhibit subtle but consistent morphological differences, particularly in conidial size, curvature, septation, and pigmentation, raising questions about phenotypic plasticity versus cryptic diversification (Linder 1929; Goos 1989; Tsui et al. 2001; Zhao et al. 2007; Lu et al. 2018a, 2018b; Yang et al. 2023; Ma et al. 2024a, 2024b, 2025; Lin et al. 2025). Our new habitat record further reveals comparable morphological divergence from the type specimen of *Neohelicomyces astrictus* across different habitats, primarily reflected in differences in conidiophore and conidial dimensions.

The habitat-associated morphological divergence highlights the limitations of morphology-based identification alone and highlights the necessity of molecular data for accurate species delimitation (Lin et al. 2025; Ma et al. 2025). Consequently, integrative taxonomic approaches integrating detailed morphological comparisons and multigene phylogenetic analyses contribute to a deeper understanding of species delimitation and evolutionary relationships within *Neohelicomyces*.

Freshwater fungi are a special ecological group that plays important roles in the decomposition of submerged organic matter and in nutrient cycling within aquatic ecosystems (Shearer et al. 2007; Jones et al. 2014; Hyde et al. 2016a; Tsui et al. 2016; Calabon et al. 2023; Xu et al. 2025). In our survey of freshwater fungi, three species of *Neohelicomyces* were identified, including two new species, *N. brevis* and *N. wujiangensis*, as well as a new habitat record of *N. astrictus*. The three species are well supported as distinct lineages based on morphological characteristics and multi-locus phylogenetic analyses (LSU, ITS, *rpb*2, and *LSU*1-α). Their occurrence in freshwater habitats further expands the known ecological range of *Neohelicomyces* and suggests adaptive capacity to aquatic environments. However, studies of freshwater fungi remain more challenging due to several factors, including difficulties in sampling and isolation, the often transient or inconspicuous nature of morphological structures under aquatic conditions, and the complexity of culturing and species delimitation (Grossart and Rojas-Jimenez 2016; Hyde et al. 2016a; Barros et al. 2024). Future studies should expand sampling across geographically and ecologically diverse freshwater systems, integrate additional molecular markers and genomic data, and further explore the ecological functions and evolutionary adaptations of *Neohelicomyces* in freshwater environments.

## Supplementary Material

XML Treatment for Neohelicomyces astrictus

XML Treatment for Neohelicomyces brevis

XML Treatment for Neohelicomyces wujiangensis

## Additional information

### Artificial Intelligence (AI) use

The authors accept full responsibility for the content of the manuscript, including the disclosure of any use of AI.

No AI tools were used in the preparation of this manuscript.

### Author ORCIDs

L-J. Zhang https://orcid.org/0000-0002-3234-6757

H-X. Liu https://orcid.org/0009-0004-9311-9779

X-J. Xiao https://orcid.org/0009-0003-8769-4534

X-F. Liu https://orcid.org/0000-0003-0100-2094

N-G. Liu https://orcid.org/0000-0002-9169-2350

Y-Z. Lu https://orcid.org/0000-0002-1033-5782

### Data availability

All of the data that support the findings of this study are available in the main text.
